# Association of Milk and Dairy Intake with Anxiety and Depressive Symptoms in Children and Adolescents: A Systematic Review and Meta-Analysis

**DOI:** 10.3390/nu18193197

**Published:** 2026-09-28

**Authors:** Dongyue Hu, Xiaoxiao Li, Yan Huang, Honglv Xu

**Affiliations:** School of Medicine, Kunming University, Kunming 650214, China; hudongyue12306@163.com (D.H.); lxx09211212@163.com (X.L.); 15278629403@163.com (Y.H.)

**Keywords:** milk and dairy, depressive symptoms, anxiety symptoms, children and adolescents, meta-analysis

## Abstract

**Background:** The prevalence of anxiety and depressive symptoms among children and adolescents has become a growing global public health concern. However, evidence regarding the association of milk and dairy intake with anxiety and depressive symptoms in children and adolescents remains inconsistent. Thus, we conducted a systematic review and meta-analysis to evaluate this association. **Methods:** PubMed, Embase, Ovid MEDLINE, Scopus, and Web of Science were systematically searched for observational studies published up to March 2026. Studies involving children and adolescents aged 6–19 years that assessed milk and dairy intake in relation to anxiety or depressive symptoms were eligible. Study quality was assessed using the National Institute of Health Quality of Observational Cohort and Cross-Sectional Studies tool. *OR* and 95% *CI* were pooled using a random-effects model. Subgroup and sensitivity analyses were conducted. The protocol was registered with PROSPERO (CRD420261328004). **Results:** A total of 10 studies were included, comprising eight cross-sectional studies and two cohort studies, with participants aged 7–19 years. A Meta-analysis of six cross-sectional studies (123,519 participants, eight effect sizes) found no significant association between dairy intake and depressive symptoms (*OR* = 0.70, 95% *CI*: 0.46–1.07, *p* = 0.10). Exploratory subgroup analyses suggested potential inverse associations among Asian children and adolescents (*OR* = 0.40, 95% *CI*: 0.17–0.94, *p* = 0.03) and for general dairy intake (*OR* = 0.39, 95% *CI*: 0.18–0.85, *p* = 0.02). Furthermore, only two individual studies reported inverse associations between milk and dairy intake and anxiety symptoms. However, these findings could not be quantitatively synthesized. **Conclusions:** Overall, milk and dairy intake was not significantly associated with depressive symptoms among children and adolescents. Exploratory subgroup analyses suggested potential inverse associations in the Asian subgroup and the general dairy intake subgroup. The evidence was limited by the predominance of cross-sectional studies, substantial heterogeneity, the small number of studies, limited geographic representation, and the inability to formally assess publication bias. Therefore, the findings should be interpreted cautiously, and further longitudinal studies are needed to clarify the temporal relationship between milk and dairy intake and mental health outcomes.

## 1. Introduction

Mental health problems have become a major global public health concern among children and adolescents [1]. The Global Burden of Disease Study 2021 reported that the prevalence of mental disorders was 14.7% among individuals aged 10–24 years [2]. Notably, anxiety and depressive symptoms are common and burdensome among children and adolescents, with increasing prevalence and earlier onset, underscoring the importance of early identification and intervention [3,4,5]. A Meta-analysis showed that the prevalence of depressive symptoms among adolescents increased from 24.0% during 2001–2010 to 37.0% during 2011–2020 [6]. Additionally, another study has reported that the prevalence of anxiety symptoms among U.S. adolescents increased from 34.1% to 44.0% between 2012 and 2018 [7]. Childhood and adolescence represent critical developmental periods characterized by rapid brain maturation, evolving emotional regulation capacities, and the establishment of lifelong dietary habits [8]. Thus, identifying modifiable risk and protective factors during this critical period is of great public health significance.

Studies have demonstrated that, dietary behaviors may influence mental health through multiple biological pathways, including the regulation of inflammation, oxidative stress, neuroplasticity, and the gut microbiota–gut–brain axis [9,10]. Emerging evidence suggests that healthy dietary patterns are associated with a lower risk of internalizing problems, such as depressive symptoms, whereas unhealthy dietary patterns may increase the risk of adverse mental health outcomes [11]. However, evidence derived from overall dietary pattern analyses is limited in its ability to disentangle the independent effects of specific food groups. Milk and dairy are essential dietary sources of nutrients among children and adolescents, providing high-quality protein, calcium, vitamin D, and B vitamins [12]. Furthermore, fermented dairy products provide probiotics and a range of bioactive components [13]. Notably, specific bioactive components in these products, such as tryptophan and vitamin D, alongside probiotics and other metabolites in fermented dairy, may play a role in regulating anxiety and depressive symptoms [14].

Although the association of milk and dairy with pediatric anxiety and depression has received considerable research attention, findings to date have been largely equivocal. Liu et al. [15] reported that habitual dairy consumption (≥6 days/week) was inversely associated with both depressive symptoms (*OR* = 0.602) and social anxiety (*OR* = 0.622). Similarly, Khanna et al. [16] found that regular milk consumption at breakfast was inversely associated with depressive symptoms (*OR* = 0.14) and anxiety symptoms (*OR* = 0.28) among boys aged 13–15 years. In contrast, other studies have reported no significant association among children and adolescents. For example, Kong et al. [17] found no association between milk intake and depressive symptoms among adolescents in the United States (*OR* = 1.08, 95% *CI*: 0.93–1.26). Similarly, Arat et al. [18] observed no significant associations among Asian Americans (*OR* = 0.80, 95% *CI*: 0.50–1.28) or African adolescents (*OR* = 1.11, 95% *CI*: 0.94–1.31). Notably, systematic reviews and Meta-analyses examining the association of milk and dairy intake with depressive symptoms remain limited. Furthermore, existing evidence is primarily based on adult populations [19,20]. However, children and adolescents represent a distinct developmental stage characterized by unique nutritional requirements, metabolic profiles, dietary behaviors, and emotion regulation processes. Consequently, findings from adult populations cannot be directly generalized to this age group [21,22]. In addition, evidence regarding the association of milk and dairy intake with anxiety symptoms in children and adolescents is particularly scarce. Therefore, this study aimed to systematically evaluate the association of milk and dairy intake with anxiety and depressive symptoms in children and adolescents by quantitatively synthesizing the available evidence through a Meta-analysis, thereby providing evidence for mental health promotion and nutritional intervention strategies in this population.

## 2. Methods

This systematic review and Meta-analysis were conducted following the Preferred Reporting Items for Systematic Reviews and Meta-Analyses (PRISMA) guidelines [23]. The PRISMA checklist is provided in Table S1. The protocol was registered with the International Prospective Register of Systematic Reviews (PROSPERO, CRD420261328004).

### 2.1. Search Strategy

A systematic literature search was conducted in PubMed, Embase, Ovid MEDLINE, Scopus, and Web of Science from database inception to March 8, 2026. The following controlled vocabulary and free-text terms were used in the literature searches: (Anxiety OR “Anxiety Disorders” OR “anxiet*” OR “anxiou*” OR Depression OR “Depressive Disorder” OR “depressi*” OR “Mental health” OR “well being” OR wellbeing) AND (Milk OR “Dairy Products” OR Yogurt OR “cow milk” OR “dairy milk” OR “milk product” OR “yogh*”) AND (Child OR children OR Adolescent OR adolescence OR “teen*” OR “youth*”). In addition, the reference lists of the included studies were manually searched to identify potentially eligible studies that were not retrieved through the database search. The detailed search strategies are provided in Table S2.

### 2.2. Eligibility Criteria

#### 2.2.1. Inclusion Criteria

Studies were included if they met the following criteria: (1) participants were children and adolescents aged 6–19 years; (2) the study was an observational design, including cohort, cross-sectional, or case–control studies; (3) studies assessed milk or dairy intake. Exposure categories were predefined according to the reported dietary exposure. Studies specifically assessing milk consumption alone were classified as milk intake. Studies assessing multiple types of dairy products without separate estimates for milk were classified as general dairy intake. Studies reporting combined consumption of milk and other related products were categorized according to the original study definition; (4) the outcomes were anxiety and/or depression, assessed by clinical diagnosis, validated psychometric instruments, or self-reported responses to structured questionnaire items; and (5) the study was published in English.

#### 2.2.2. Exclusion Criteria

Studies were excluded if they met any of the following criteria: (1) animal experiments, cell studies, reviews, letters, conference abstracts, case reports, or interventional studies aimed at symptom improvement (e.g., randomized controlled trials (RCTs)); (2) studies not relevant to the research topic; (3) studies focused exclusively on flavored milk, ice cream, or butter; or (4) duplicate publications based on the same cohort or survey data, only the publication with the largest sample size or the most complete data was included.

### 2.3. Study Selection

Study selection was conducted using a two-stage screening process. Two reviewers (LXX and HY) independently screened all records. EndNote 21 and Zotero 9 were used for reference management. In the first stage, duplicates were removed, and titles and abstracts were screened to exclude irrelevant studies. In the second stage, full-text articles of potentially eligible studies were assessed for inclusion. Any disagreements arising at either stage were resolved by discussion with a third reviewer (HDY).

### 2.4. Data Extraction

Data were independently extracted by two reviewers (LXX and HY). The extracted information included first author and year, country, study design, participants’ age, assessment methods for anxiety and depressive symptoms, type and assessment method of dairy intake, main findings, and adjusted confounders. Any disagreements were resolved through discussion with a third reviewer (HDY).

### 2.5. Study Quality Assessment

The quality of the included studies was assessed by three independent reviewers (HDY, HY, and LXX) using the National Institute of Health Quality of Observational Cohort and Cross-Sectional Studies tool [24]. The tool consists of 14 items, with each item rated as “Yes,” “No,” or “Other” (cannot determine, not applicable, or not reported). Overall study quality was rated based on the number of “Yes” responses and classified as good (10–14), fair (6–9), or poor (0–5). Any discrepancies among the reviewers were resolved through discussion to reach a consensus.

### 2.6. Statistical Analysis

The associations of milk and dairy intake with anxiety and depressive symptoms were summarized using odds ratios (*OR*) with corresponding 95% confidence intervals (95% *CI*). When necessary, effect estimates were calculated or transformed from published data. When studies reported multiple exposure categories, a representative exposure category reflecting a commonly consumed level was selected while avoiding extreme categories. A random-effects model was used to account for potential heterogeneity. Heterogeneity was assessed using the *I*^2^ statistic, with values of ≤25%, 25–50%, and >50% indicating low, moderate, and high heterogeneity, respectively [25]. Subgroup analyses were conducted based on geographic region of the study population and dairy type to explore potential sources of heterogeneity. Sensitivity analyses were performed using a leave-one-out approach to assess the robustness of the pooled results [26]. A two-sided *p* < 0.05 was considered statistically significant. All analyses were performed using Review Manager (RevMan) version 5.4.1.

## 3. Results

### 3.1. Characteristics of Included Studies

A total of 2503 records were retrieved from five databases, of which 682 were duplicates. After title and abstract screening of the remaining 1821 records, 47 full-text articles were assessed for eligibility based on the predefined criteria. Ultimately, 10 studies were included in the systematic review [15,16,17,18,27,28,29,30,31,32]. Figure 1 illustrates the study selection process.

In the 10 included studies, eight were cross-sectional studies [15,16,17,18,27,28,29,32] and two were cohort studies [30,31]. The studies were conducted in China (*n* = 2), the United States (*n* = 2), Korea (*n* = 1), India (*n* = 1), Japan (*n* = 1), Canada (*n* = 2), and Mexico (*n* = 1). The study populations consisted of children and adolescents aged 7–19 years. Table 1 presents the general characteristics of the included studies, while Table S3 summarizes the results of the quality assessment. The quality assessment indicated that two studies were of good quality and eight were of moderate quality.

Six cross-sectional studies, comprising a total of 123,519 participants, were included in the Meta-analysis. These studies provided *OR* with corresponding 95% *CI* for the association of milk and dairy intake with depressive symptoms [15,16,17,18,27,28]. Arat et al. [18] reported three independent effect estimates stratified by race (Asian Americans, Caucasian, and African). Although Khanna et al. [16] included both boys and girls, only the estimate for boys was available because the corresponding data for girls were not reported. Accordingly, eight effect sizes were pooled in the Meta-analysis. Tanaka et al. [32] reported Pearson’s correlation coefficient (*r*), and Sámano et al. [29] reported adjusted prevalence ratios (*aPRs*). These effect measures were not directly comparable with *OR* and therefore could not be pooled in the Meta-analysis. In addition, because only two cohort studies were identified [30,31], these studies were synthesized qualitatively.

### 3.2. Meta-Analysis of the Association of Milk and Dairy Intake with Depressive Symptoms in Children and Adolescents

#### 3.2.1. Overall Pooled Analysis

Figure 2 presents the overall pooled estimate for the association of milk and dairy intake with depressive symptoms among children and adolescents. Among the eight cross-sectional studies included in the systematic review, six studies with sufficient data for quantitative synthesis were included in the Meta-analysis, comprising 123,519 participants and eight effect sizes. Using a random-effects model, the pooled analysis showed no statistically significant association between milk and dairy intake and depressive symptoms (*OR* = 0.70, 95% *CI*: 0.46–1.07, *p* = 0.10, *I*^2^ = 98%).

#### 3.2.2. Subgroup Analyses

Given the high between-study heterogeneity observed in the overall pooled analysis, exploratory subgroup analyses were conducted to explore potential sources of this heterogeneity. Figure 2 presents the results stratified by geographic region. Within the Asian subgroup (comprising populations from India, Korea, and China), an exploratory analysis suggested a potential inverse association (*OR* = 0.40, 95% *CI*: 0.17–0.94, *p* = 0.03, *I*^2^ = 99%). In contrast, no statistically significant association was observed within the Americas subgroup (*OR* = 1.16, 95% *CI*: 0.92–1.46, *p* = 0.21, *I*^2^ = 83%). The between-subgroup difference was statistically significant (*p* = 0.02).

Figure 2 presents the results stratified by dairy type. Exploratory analyses suggested a potential inverse association for general dairy intake (*OR* = 0.39, 95% *CI*: 0.18–0.85, *p* = 0.02, *I*^2^ = 90%). Conversely, no statistically significant association was observed regarding exclusive milk intake and depressive symptoms (*OR* = 0.97, 95% *CI*: 0.75–1.25, *p* = 0.80, *I*^2^ = 93%). This between-subgroup difference was statistically significant (*p* = 0.03).

#### 3.2.3. Sensitivity Analysis and Publication Bias

Table 2 presents the results of the leave-one-out sensitivity analysis. Sequential exclusion of individual studies did not materially alter the direction or magnitude of the pooled association between milk and dairy intake and depressive symptoms. The recalculated pooled *OR* ranged from 0.62 to 0.92, and all estimates remained below 1.00, suggesting a consistent direction of association. Although excluding Ding et al. [28] slightly reduced heterogeneity (*I*^2^ from 98% to 92%), substantial heterogeneity remained after excluding any individual study. These findings suggest that no single study had a substantial influence on the overall pooled estimate.

Given the limited number of included studies, Egger’s test was not performed because its statistical power was insufficient. Therefore, publication bias was not quantitatively assessed.

### 3.3. Association of Milk and Dairy Intake with Anxiety Symptoms

Given that only two cross-sectional studies reported the association of milk and dairy intake with anxiety symptoms, a quantitative Meta-analysis was not conducted. Khanna et al. [16] found that consuming milk at breakfast was inversely associated with anxiety symptoms among Indian male adolescents (*OR* = 0.28, 95% *CI*: 0.10–0.77). Liu et al. [15] reported that habitual dairy consumption (≥6 days/week) was inversely associated with social anxiety symptoms in Chinese children and adolescents (*OR* = 0.622, 95% *CI*: 0.418–0.923).

## 4. Discussion

In this systematic review and Meta-analysis, we quantitatively assessed the association of milk and dairy intake with anxiety and depressive symptoms in children and adolescents. Six cross-sectional studies comprising 123,519 participants were included in the quantitative synthesis. Overall, no statistically significant association was observed between milk and dairy intake and depressive symptoms. However, subgroup analyses revealed that the association may vary according to geographic region and the type of dairy intake. Exploratory subgroup analyses suggested a potential inverse association between milk and dairy intake and depressive symptoms among Asian populations. Exploratory analyses by exposure type also suggested a potential inverse association for general dairy intake, whereas milk intake alone showed no statistically significant association. Furthermore, only two studies examined anxiety symptoms, both suggesting an inverse association with milk and dairy intake. However, the limited number of studies precluded a quantitative Meta-analysis. Overall, current evidence does not support a definitive association between milk and dairy intake and depressive symptoms, while subgroup findings may provide directions for future research.

Our findings are largely consistent with previous Meta-analyses conducted in adults. For instance, a systematic review of 13 observational studies found that while one study identified a positive association and five showed no association, seven yielded mixed findings dependent on dairy type, sex, or population characteristics [19]. Furthermore, a Meta-analysis of eight cohort studies demonstrated that fermented dairy intake was associated with a reduced risk of depressive symptoms (*OR* = 0.89) [20]. Compared with adults, children and adolescents have distinct metabolic characteristics, gut microbiota composition, and dietary behavior patterns [22]. Therefore, the present study contributes to the limited evidence regarding the potential relationship between dairy intake and mental health outcomes in children and adolescents [33]. Beyond depressive symptoms, prior research has devoted relatively little attention to anxiety. Only two cross-sectional studies reported data on anxiety symptoms, both of which were conducted in Asian countries (India and China). Evidence on the association between milk and dairy intake and anxiety symptoms in children and adolescents remains scarce, and further well-designed studies are warranted to validate these findings.

Although the overall analysis showed no significant association between milk and dairy intake and depressive symptoms, exploratory subgroup analyses suggested a potential inverse association for general dairy intake. Several biological mechanisms may plausibly contribute to the observed associations; however, these mechanisms remain speculative and cannot establish causality. First, dairy provides essential tryptophan [14], and its α-lactalbumin facilitates tryptophan transport across the blood–brain barrier to enhance 5-hydroxytryptamine (5-HT) synthesis and modulate stress [34,35,36]. Second, dairy-derived vitamin D and calcium [12,37] synergistically support neural regulation. Vitamin D facilitates monoamine neurotransmitter synthesis [38,39,40] and maintains calcium homeostasis for synaptic plasticity [41]. Furthermore, it modulates the hypothalamic–pituitary–adrenal (HPA) axis to buffer stress responses [42,43]. Finally, compared to conventional milk, fermented dairy products (e.g., yogurt and cheese) supply not only protein and calcium but also probiotics and diverse bioactive metabolites [44]. This enhances emotional well-being via the gut–brain axis by regulating inflammation, immunity, and neuroactive metabolism [45,46,47,48,49]. Nevertheless, these mechanistic interpretations should be considered hypotheses requiring further validation, as direct evidence linking dairy intake, biological pathways, and mental health outcomes remains limited.

Exploratory subgroup analyses suggested potential regional differences in the association between dairy intake and depressive symptoms, although the overall pooled estimate was not statistically significant. Dairy intake showed a potential inverse association with depressive symptoms among Asian adolescents, whereas no statistically significant association was observed among those in the Americas. Several factors may contribute to these regional differences. First, dairy consumption habits and types vary across regions. Because traditional Asian diets typically feature low dairy intake, individuals with higher consumption often belong to families with higher socioeconomic status or greater health consciousness. These confounding factors might contribute to the observed protective effect [50,51,52]. Second, genetic backgrounds and metabolic differences may play a role. For example, the distribution of lactase persistence genes varies across populations. This genetic variation affects dairy absorption and subsequent gut responses [53]. In addition, differences in overall dietary patterns, cultural background, and the assessment of anxiety and depressive symptoms may also contribute to the inconsistent findings across regions. Collectively, these findings highlight that the association of dairy intake with mental health may be highly population-specific. Future studies across diverse cultural backgrounds are necessary to verify these findings.

A primary limitation of this Meta-analysis is the substantial between-study heterogeneity. Although subgroup analyses indicated that geographic region and dairy type may partly explain the observed heterogeneity, heterogeneity remained high within subgroups. This suggests the presence of other unidentified influencing factors. This heterogeneity may be related to differences in exposure definitions, outcome assessment methods, study populations, and the extent of confounder adjustment. Specifically, the included studies varied considerably in the covariates considered in multivariable analyses, with differences in the adjustment for sociodemographic characteristics, lifestyle behaviors, and other potential confounding factors. Such variability in confounder control may have influenced the estimated associations and contributed to residual confounding across studies. Additionally, variations in sample sources, age demographics, and regional backgrounds could further widen these between-study differences. Therefore, the pooled estimates should be interpreted cautiously, as they may reflect both true variability in associations and methodological differences across studies. Future studies using standardized exposure definitions, validated outcome assessment methods, and more consistent adjustment strategies are warranted to clarify these associations.

There are several strengths to the present study. First, to our knowledge, this is the first Meta-analysis to evaluate the association of milk and dairy intake with depressive and anxiety symptoms in children and adolescents. Second, we distinguished milk intake from general dairy intake, enabling the evaluation of potential differences according to exposure definition. Third, exploratory subgroup analyses suggested that geographic region and dairy type may be important sources of heterogeneity, indicating that the association between dairy intake and mental health may be context-dependent. Finally, this study summarized the currently available evidence regarding anxiety symptoms. Although only limited evidence was available and quantitative synthesis was not feasible, these findings highlight the need for further research investigating the relationship between milk and dairy intake and anxiety symptoms. However, several limitations must be acknowledged. First, all included studies utilized a cross-sectional design, which precludes causal inference and raises the possibility of reverse causality. Second, substantial differences in exposure definitions, outcome assessment, and adjustment for confounding across studies may have contributed to the observed heterogeneity. In particular, variations in the definitions of milk and dairy intake across studies may have limited direct comparability between exposure categories. Third, RCTs and other intervention studies were not included, and thus the potential effects of modifying milk and dairy intake on anxiety and depressive symptoms could not be assessed. Fourth, publication bias could not be assessed because fewer than 10 studies were available for each Meta-analysis. Although this approach is consistent with current recommendations, potential small-study effects cannot be excluded, particularly considering the limited number of available studies. Fifth, some subgroup analyses included multiple effect estimates derived from the same study, which may have reduced the statistical independence of estimates and potentially influenced subgroup findings. Sixth, the geographic representation of the included evidence was limited, as all studies were conducted in Asia and North America, with no available data from Europe, South America, Africa, or Oceania. Therefore, the pooled estimates may not be generalizable to children and adolescents from underrepresented regions. Finally, although every effort was made to identify all relevant studies, the evidence on anxiety symptoms remains limited, and the available studies are insufficient to support a reliable quantitative Meta-analysis.

## 5. Conclusions

This Meta-analysis found no overall association between milk and dairy intake and depressive symptoms in children and adolescents. However, subgroup analyses suggested potential inverse associations for general dairy intake and among Asian populations. These findings should be interpreted cautiously due to substantial heterogeneity, the limited number of available studies, possible small-study effects, and residual confounding related to socioeconomic and lifestyle factors. Evidence on anxiety symptoms was too limited to allow quantitative synthesis. Prospective cohort studies are needed to clarify the temporal relationship between milk and dairy intake and mental health outcomes and to explore potential variations by dairy type and geographic region.

## Figures and Tables

**Figure 1:**
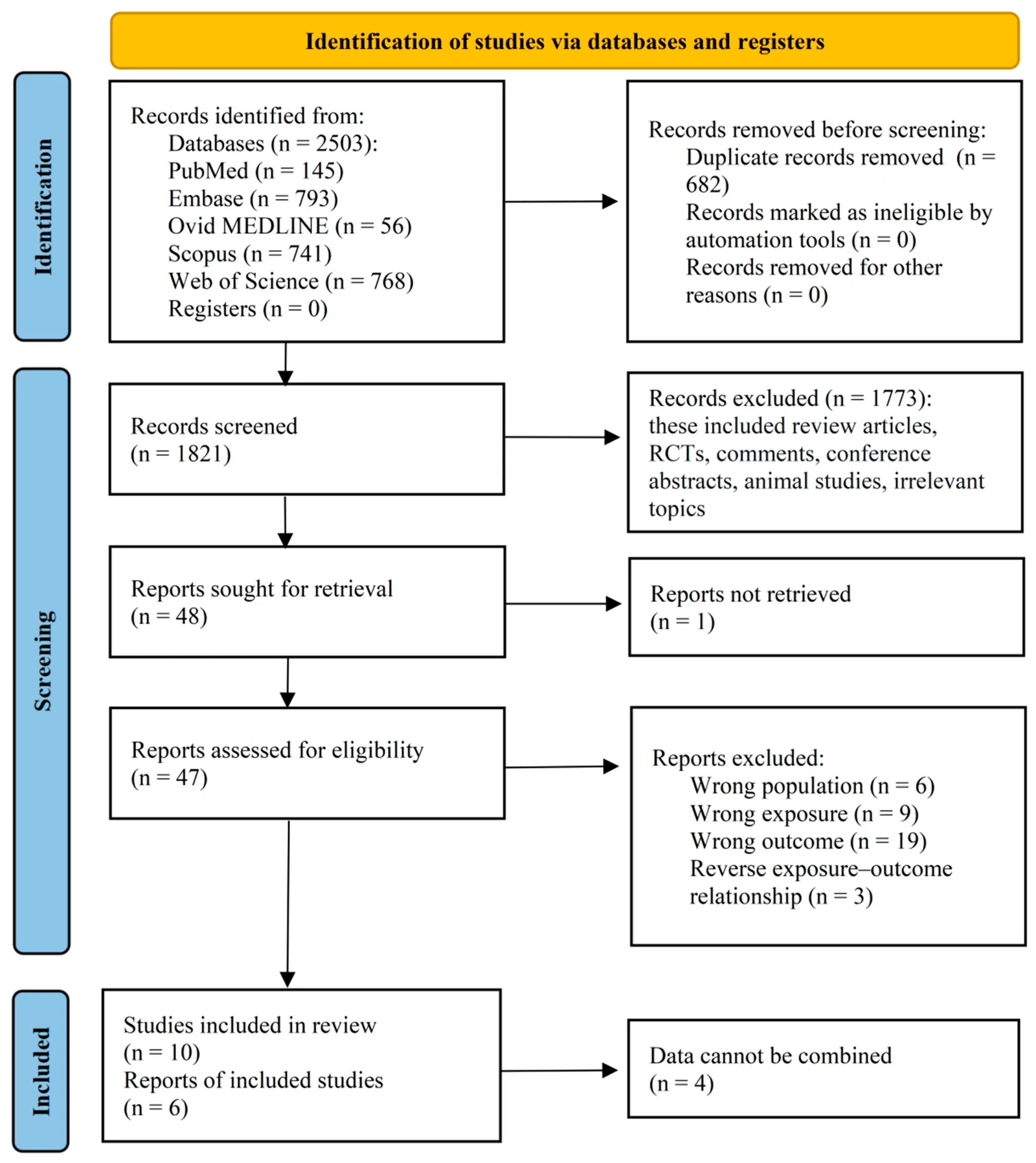
Flow chart of study selection.

**Figure 2:**
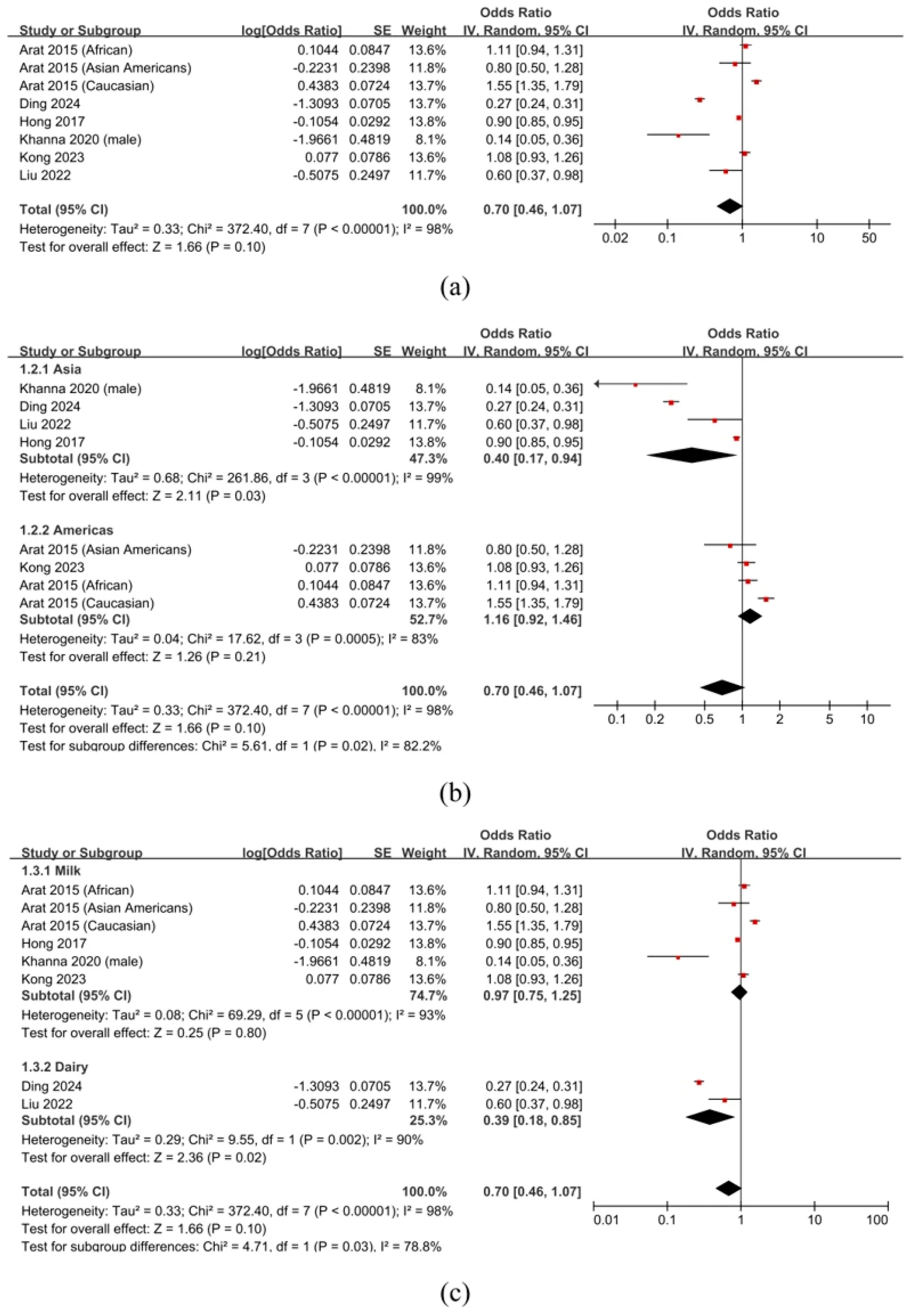
Forest plots of the association between milk and dairy intake and depressive symptoms. (**a**) Overall pooled analysis [15,16,17,18,27,28]; (**b**) subgroup analysis stratified by geographic region [15,16,17,18,27,28]; (**c**) subgroup analysis stratified by dairy type [15,16,17,18,27,28].

**Table 1:** Characteristics of included studies.

Author, Year	Country	Study Design	Sample Size	Age (Range/Mean ± SD)	Outcome Assessment	Exposure Assessment	Main Findings	Adjustment of Confounders
Khanna, 2020 [16]	India	Cross-sectional	546 (males: 325; females: 221)	13–15	**Anxiety symptoms** **Depressive symptoms** Parent-reported (CBCL)	**Milk** (24 h recall and food frequency questionnaire)	**Male: depressive symptoms** (***OR* = 0.14, 95% *CI*: 0.05–0.36**); anxiety symptoms (*OR* = 0.28, 95% *CI*: 0.10–0.77). Female: depressive symptoms (*OR* not reported).	Multivariable regression analysis; covariates not reported.
Kong, 2023 [17]	United States	Cross-sectional	13,677	12–18	**Depressive symptoms** Self-reported (single-item YRBS question)	**Milk** Self-reported (YRBS)	***OR* = 1.08, 95% *CI*: 0.93–1.26.**	Sex, age, grade, race, overweight, obesity.
Hong, 2017 [27]	Korea	Cross-sectional	65,212	12–18	**Depressive symptoms** Self-reported (single-item measure)	**Milk** Self-reported (KYRBS)	1–2 times/week: *OR* = 0.93, 95% *CI*: 0.88–0.98; 3–4 times/week: *OR* = 0.93, 95% *CI*: 0.88–0.99; 5–6 times/week: *OR* = 0.89, 95% *CI*: 0.84–0.95; **once/day: *OR* = 0.90, 95% *CI*: 0.85–0.96**; twice/day: *OR* = 1.02, 95% *CI*: 0.94–1.11; ≥3 times/day: *OR* = 1.06, 95% *CI*: 0.96–1.17.	Sex, age, school level, school types, socioeconomic status, place of residence, BMI, physical activity, smoking, and alcohol drinking.
Arat, 2015 [18]	United States	Cross-sectional	Total: 10,563 (Asian: 671; Caucasian: 6415; African: 3477)	12–18	**Depressive symptoms** Self-reported (Single item)	**Milk** Self-reported (YRBS)	**Caucasian students: *OR* = 1.55, 95% *CI*: 1.34–1.78.** **Asian Americans students: *OR* = 0.80, 95% *CI*: 0.50–1.28.** **African students: *OR* = 1.11, 95% *CI*: 0.94–1.31.**	Bivariate logistic regression; no covariates were adjusted for.
Liu, 2022 [15]	China	Cross-sectional	1353	7–17	**Depressive symptoms** Self-reported (CDI) **Social anxiety symptoms** Self-reported (SASC)	**Dairy** Self-reported	**Depressive symptoms: *OR* = 0.602, 95% *CI*: 0.369–0.982**; social anxiety symptoms: *OR* = 0.622, 95% *CI*: 0.418–0.923.	Age, sex, parental educational attainment, single-child status, BMI, income, consumption of fruit, SSB, fried food, and physical activity.
Dabravolskaj, 2023 [30]	Canada	Prospective cohort	24,274	Baseline: 14.8 ± 1.2 years Follow-up: 15.8 ± 1.2 years	**Depressive symptoms** Self-reported (CESD-R-10) Anxiety symptoms Self-reported (GAD-7)	**Milk and alternatives** Self-reported (COMPASS)	Depressive symptoms: *β* = −0.03, 95% *CI*: −0.16–0.10; anxiety symptoms: *β* = 0.08, 95% *CI*: −0.04–0.19.	Age, sex, ethnicity, school-area urbanicity, school-area median household income, weight status, weight perception, weight loss attempts, and baseline mental health; mutually adjusted for all other lifestyle behaviours.
Tanaka, 2019 [32]	Japan	Cross-sectional	Total: 858 (junior high school students: 441; senior high school students: 417)	15.49 ± 1.78 years	**Depressive symptoms** Self-reported (CESD-20)	**Milk and dairy products** Self-reported (Japanese Food Guide Spinning Top)	Junior high school students: *r* = −0.02, 95% *CI*: −0.11–0.07. Senior high school students: *r* = −0.03, 95% *CI*: −0.12–0.07.	Age, sex, and sleep duration.
Loewen, 2019 [31]	Canada	Prospective cohort	3436	Baseline: 10–11 Follow-up: 14–15	**Mental illness** (internalizing, externalizing, and other psychiatric disorders) Clinical diagnosis (ICD-9/10)	**Milk and alternatives** self- and parent-reported (Harvard Youth/Adolescent Food Frequency Questionnaire)	Overall: *RR* = 0.83, 95% *CI*: 0.66–1.05. Boys: *RR* = 0.65, 95% *CI*: 0.48–0.88. Girls: OR not reported (association not significant).	Sex, weight status, parental education, household income, residential area, energy intake, fruit and vegetable intake, grain intake, meat and alternatives intake, saturated fat, added sugar, physical activity, sleep, and screen time.
Ding, 2024 [28]	China	Cross-sectional	32,389	Grades 4–12	**Depressive symptoms** Self-reported (CESD-20)	**Milk/yogurt/soy milk** self-reported (lifestyle questionnaire)	1 day/week: *OR* = 0.70, 95% *CI*: 0.59–0.85; 2 days/week: *OR* = 0.79, 95% *CI*: 0.67–0.93; 3 days/week: *OR* = 0.65, 95% *CI*: 0.55–0.76; 4 days/week: *OR* = 0.50, 95% *CI*: 0.42–0.59; 5 days/week: *OR* = 0.44, 95% *CI*: 0.38–0.52; 6 days/week: *OR* = 0.43, 95% *CI*: 0.36–0.51; **7 days/week: *OR* = 0.27, 95% *CI*: 0.24–0.31**.	Grade, sex, and urban/rural area.
Sámano, 2026 [29]	Mexico	Cross-sectional	344	10–19	**Depressive symptoms** Self-reported (EPDS)	**Milk and dairy products** self-reported (FFQ)	*aPR* = 1.78, 95% *CI*: 1.16–2.73.	Age, marital status, socioeconomic status, educational level, educational lag, pre-pregnancy BMI, sleep duration, and physical activity.

Effect estimates shown in bold in the “Main findings” column represent the results included in the Meta-analysis.

**Table 2:** Sensitivity analysis of the association between milk and dairy intake and depressive symptoms.

Omitted Study	Pooled *OR* (95% *CI*)	*I*^2^ (%)	*p*
None (All studies)	0.70 (0.46, 1.07)	98	0.10
Arat 2015 (African) [18]	0.65 (0.40, 1.05)	98	0.08
Arat 2015 (Asian Americans) [18]	0.69 (0.44, 1.08)	98	0.10
Arat 2015 (Caucasian) [18]	0.62 (0.39, 0.98)	98	0.04
Ding 2024 [28]	0.92 (0.72, 1.17)	92	0.49
Hong 2017 [27]	0.65 (0.35, 1.20)	98	0.17
Khanna 2020 (male) [16]	0.81 (0.53, 1.24)	98	0.33
Kong 2023 [17]	0.65 (0.40, 1.06)	98	0.08
Liu 2022 [15]	0.72 (0.46, 1.12)	98	0.14

## Data Availability

The original contributions presented in this study are included in the article. Further inquiries can be directed to the corresponding author.

## Supplementary Materials

The following supporting information can be downloaded at: https://www.mdpi.com/article/10.3390/nu18193197/s1, Table S1. PRISMA Checklist; Table S2. Search strategy and the results of literature searching; Table S3. The results of the quality evaluation of the included studies.
